# Multi-lectin Affinity Chromatography and Quantitative Proteomic Analysis Reveal Differential Glycoform Levels between Prostate Cancer and Benign Prostatic Hyperplasia Sera

**DOI:** 10.1038/s41598-018-24270-w

**Published:** 2018-04-25

**Authors:** Sarah M. Totten, Ravali Adusumilli, Majlinda Kullolli, Cheylene Tanimoto, James D. Brooks, Parag Mallick, Sharon J. Pitteri

**Affiliations:** 1 Canary Center at Stanford for Cancer Early Detection, Department of Radiology, Stanford University School of Medicine, Palo Alto, CA 94304 USA; 2 Department of Urology, Stanford University School of Medicine, Stanford, CA 94305 USA

## Abstract

Currently prostate-specific antigen is used for prostate cancer (PCa) screening, however it lacks the necessary specificity for differentiating PCa from other diseases of the prostate such as benign prostatic hyperplasia (BPH), presenting a clinical need to distinguish these cases at the molecular level. Protein glycosylation plays an important role in a number of cellular processes involved in neoplastic progression and is aberrant in PCa. In this study, we systematically interrogate the alterations in the circulating levels of hundreds of serum proteins and their glycoforms in PCa and BPH samples using multi-lectin affinity chromatography and quantitative mass spectrometry-based proteomics. Specific lectins (AAL, PHA-L and PHA-E) were used to target and chromatographically separate core-fucosylated and highly-branched protein glycoforms for analysis, as differential expression of these glycan types have been previously associated with PCa. Global levels of CD5L, CFP, C8A, BST1, and C7 were significantly increased in the PCa samples. Notable glycoform-specific alterations between BPH and PCa were identified among proteins CD163, C4A, and ATRN in the PHA-L/E fraction and among C4BPB and AZGP1 glycoforms in the AAL fraction. Despite these modest differences, substantial similarities in glycoproteomic profiles were observed between PCa and BPH sera.

## Introduction

Prostate cancer (PCa) is one of the most common cancers among men in the U.S., with a projected 161,360 new cases in 2017 and an estimated 26,730 prostate cancer deaths^1^. For nearly three decades, prostate-specific antigen (PSA) has been used for prostate cancer screening, resulting in a significant increase in the number of detected cases of prostate cancer, with a shift toward detecting the cancer at earlier stages^2,3^. The beneficial effects of PSA screening are still debated, as conflicting evidence has been presented regarding whether or not it reduces prostate cancer mortality rates^3–7^. Circulating levels of PSA are also affected by other conditions of the prostate, including certain infections and inflammation, such as prostatitis, and benign enlargement of the prostate (benign prostatic hyperplasia, or BPH)^8^. Due to this lack of specificity for prostate cancer, the diagnostic capability of PSA suffers from a high number of false positives, resulting in unnecessary biopsies and overdiagnosis. Therefore there is a clinical need for a biomarker with greater specificity for prostate cancer that can distinguish between patients with benign disease from those at higher risk for prostate cancer, allowing patients to receive appropriate treatment and thus reducing the number of unnecessary biopsies, invasive surgeries, and the associated side effects.

The blood contains thousands of circulating proteins that are reflective of physiological and pathological states within the body, providing a rich source of potential biomarkers that can be easily sampled through less invasive means. However, performing in-depth plasma or serum proteomics is analytically challenging due to the complexity of the protein mixture and the large dynamic range of protein concentrations in the blood, which spans more than ten orders of magnitude^9^. This challenge has been met with a number of chromatography and mass spectrometry-based approaches designed to systematically achieve greater coverage deeper into the plasma/serum proteome, ranging from the selection of specific subsets of proteins through affinity chromatography, to extensive pre-fractionation at the protein and peptide level^10^. The depth of proteomic analysis has also been enhanced by technological improvements in high-performance mass spectrometers with greater scan speeds, resolving power, and sensitivity. Furthermore, it is estimated that over 50% of the human proteome is glycosylated^11^. Glycosylation is a common yet highly complex post-translational modification recognized to play an important role in a variety of biological processes, such as cell-cell communication, host-pathogen interactions, and immune response^12–14^. Glycoproteins can have multiple sites of glycosylation with varying degrees of occupancy. Additionally, a variety of different glycans can occupy a given glycosylation site, giving rise to the complex microheterogeneity that notoriously complicates the characterization and analysis of protein glycosylation. Changes in glycosylation have been correlated to disease status in a variety of cancers, including prostate cancer, and exploiting these aberrancies has shown promise for use as effective biomarkers^13,15–18^. The bulk of the research on glycosylation changes in prostate cancer has focused on characterizing the various glycoforms of PSA to improve its clinical utility^19–23^. These studies find that core-fucosylation and the sialic acid linkage of PSA glycoforms play a key role in differentiating non-PCa patients and those with BPH from low- and high-risk PCa cases. A number of glycomic and glycoproteomic studies have also looked beyond PSA for prostate cancer-specific glycosylation changes in a variety of clinical samples, including urine, seminal fluid, blood plasma/serum and tissue^24^. In a review by Drake *et al*., it was reported that an increase in the expression of N-acetylglucosaminyl transferase V in prostate tumors leads to a subsequent increase in β1–6 branching, forming larger tri- and tetraantennary N-linked structures in prostate tissue^24^. Additionally, a recent review describing the mechanisms and clinical implications of altered glycosylation in cancer reports that an increases in glycan branching, as well as increased fucosylation and sialylation, are the most widely occurring cancer-associated alterations in protein glycosylation^13^. Although progress has been made in realizing the importance of glycosylation in the development and progression of cancer, it still remains a significant analytical challenge to obtain detailed glycan characterization while still retaining protein- and site-specific, information in large, complex biological mixtures derived from clinical samples.

In this study, we perform a quantitative glycoproteomic analysis using multi-lectin affinity chromatography (M-LAC) to compare the circulating levels of proteins and their glycoforms from the sera of men with BPH to those with prostate cancer. We use a series of chromatographic separations to simultaneously decomplex the protein mixture and to enrich for proteins with specific types of glycosylation by using lectins with affinity for specific glycan motifs. Here we chose *Aleuria aurantia* lectin (AAL) to capture core-fucosylated proteins and *Phaseolus vulgaris* leucoagglutinin erythroagglutinin, and *Phaseolus vulgaris* erythroagglutinin (jointly abbreviated as PHA-L/E) to capture highly branched glycans^25^. These lectins were chosen to target types of glycosylation reported to be aberrant in prostate cancer, while simultaneously fractionating the complex mixture of proteins to enhance depth of analysis. Using an M-LAC approach to separate glycoforms allows for a systematic way to screen for changes in glycosylation in a complex mixture while retaining protein-specific information. We identify differences at the global protein level as well as among specific glycoforms of quantitated proteins that could be used to aid in differentiating BPH from PCa cases.

## Methods

### Methods

#### Serum Sample Collection from Benign Prostatic Hyperplasia and Prostate Cancer Patients

De-identified serum samples used in this study were taken from an existing serum bank collected on patients immediately prior to surgery for prostate cancer, or from men with elevated serum PSA levels, known BPH, and two or more previous negative prostate biopsies. Following informed consent, all blood samples were collected in red top tubes, allowed to clot, and centrifuged. Serum was then aliquoted (500 μL) into tubes and frozen at −80 °C to limit effects of freeze/thaw cycles. Samples were retrieved and analyzed. All ten PCa samples were from men in whom the cancer volume was 1 cc or greater and showed pathological Gleason scores of 4 + 3 = 7 (Table 1). Use of the existing serum resource has been reviewed and certified by the Stanford University Institutional Review Board (IRB). The approved IRB protocol for blood collection allows for correlation of clinical information, including disease status and follow-up, with molecular measurements in the blood, including protein analysis. The patients have provided informed consent allowing for use of their tissues/blood specimens. Samples collected prior to 1999 are considered existing samples in an established clinical and tissue database, and can be used under an IRB approved Waiver.

**Table 1:** Clinical Characteristics of PCa and BPH samples.

Sample Type & Number	Age	PSA (ng/mL)	Percent G4/5*	Total Cancer Volume (cc)
PCa_1	56	10.20	70	4.37
PCa_2	66	3.92	90	8.55
PCa_3	50	15.48	80	9.00
PCa_4	58	6.36	60	9.03
PCa_5	60	21.30	90	4.93
PCa_6	70	3.27	70	6.00
PCa_7	46	30.13	90	1.00
PCa_8	68	16.11	95	29.39
PCa_9	64	13.40	60	7.20
PCa_10	56	8.71	50	4.56
BPH_1	62	3.14	N/A	
BPH_2	73	4.20		
BPH_3	69	11.16		
BPH_4	71	13.80		
BPH_5	56	7.67		
BPH_6	44	3.42		
BPH_7	61	8.21		

*Percentage of Gleason Pattern 4 or 5. The remainder is pattern 3. All PCa samples are Gleason 4 + 3 = 7.

#### Immunodepletion of Abundant Proteins

Pooled normal human male EDTA plasma was purchased from Innovative Research and used as a reference sample pool. 200 µL of each clinical serum sample (17 in total) and 17–200 µL aliquots of the reference pool were immunodepleted using CaptureSelect^TM^ HumanPlasma 14 affinity resin for the removal of abundant proteins (albumin, IgG, IgM, IgA, IgE, IgD, free light chains, transferrin, fibrinogen, α-1-antitrypsin, apolipoprotein A1, α-1-2-macroglobulin, α-1-acid-glycoprotein, and haptoglobin) as previously described^26^. The flow-through fractions from the CaptureSelect column containing the unbound proteins were desalted and concentrated using Amicon Ultra 15 mL 3 K NMWL centrifugal filters (Millipore). An aliquot of 10 µL from each concentrated sample was set aside for a Bradford assay to determine total protein concentration, and the remainder of the sample was diluted 1:10 in protein denaturation buffer (8 M urea, 50 mM Tris-HCl, 0.05% octyl β-D-glucopyranoside, pH 7.5, prepared in 100 mM ammonium bicarbonate).

#### Reduction, Alkylation, and Isotopic Labeling

Protein disulfide bonds were reduced using dithiothreitol (DTT) at a final concentration of 5.5 mM for two hours at room temperature. Cysteine residues were alkylated using acrylamide for one hour at room temperature in the dark. For relative quantitation, all clinical BPH and PCa samples were alkylated with 1,2,3-^13^C3 (heavy) acrylamide in a 7.4 mg per mg of protein ratio, and all reference samples were alkylated with 1,2,3-^12^C3 (light) acrylamide in a 7.1 mg per mg of protein, as described in Faca *et al*.^27^. After alkylation, each clinical sample was then combined with a reference sample, making 7 BPH/Reference pairs and 10 PCa/Reference pairs. Each pair was concentrated and buffer exchanged into 1 mL of 1 × PBS for subsequent lectin chromatography.

#### Multi-Lectin Affinity Chromatography

Multi-lectin affinity chromatography (M-LAC) was used to separate proteins by specific glycoforms. The following M-LAC experiments were modified for use on this specific application from previously described methodologies employing immunodepletion and M-LAC to investigate glycoproteomic changes in human plasma and serum^28–32^. *Aleuria aurantia* lectin (AAL), *Phaseolus vulgaris* leucoagglutinin, and *Phaseolus vulgaris* erythroagglutinin (PHA-L/E) were used to capture core fucosylated protein glycoforms, and glycoforms carrying highly branched complex type glycans, respectively. Agarose-bound AAL and PHA-L/E lectins were purchased from Vector Laboratories (Burlingame, CA) and gravity packed in house, as previously described^25^. All chromatography was performed on an Agilent 1260 Bio-Inert HPLC system, equipped with a quaternary pump, a manual injector, UV multiple wavelength detector, and an analytical-scale fraction collector. Reduced and alkylated protein was loaded onto the M-LAC column for fractionation. First the flow-through fraction was collected, containing the non- or otherwise-glycosylated proteins making up the unbound (UNB) fraction. Bound glycoproteins were eluted in series using competitive saccharide binding and low pH elution buffers. Core-fucosylated glycoforms bound to AAL were eluted with 200 mM L-fucose ACROS Organics). Lastly, glycoforms bound to PHA-L/E were eluted with 100 mM acetic acid, pH 3.8. Each of the three M-LAC fractions (henceforth abbreviated as UNB, AAL, and PHA) were concentrated to 250 µL using 3 K NMWL Amicon centrifugal filters.

#### Reversed-Phase Chromatography

Reversed-phase (RP) fractionation was performed on a 100 mm × 2.1 mm ID stainless steel column packed with POROS^®^R2 (Applied Biosystems), with a 2,000 Å particle size, poly styrene-divinylbenzene stationary phase. Each M-LAC fraction was further separated into 13 RP fractions using an increasing gradient of organic mobile phase as follows: 0–5 minutes at 100% buffer A (0.1% trifluoroacetic acid in water); 5–38 minutes, ramp to 90% buffer B (0.1% trifluoroacetic acid in acetonitrile); 38–40 minutes hold at 90% buffer B; 40–50 minutes hold at 95% buffer A for re-equilibration. In total, 39 fractions (13 RP fractions x 3 M-LAC fractions) were collected per patient sample. All fractions were frozen at −80 °C overnight and lyophilized. RP fractions were reconstituted in 50 µL of 50 mM ammonium bicarbonate in 4% acetonitrile and subsequently digested with 0.5 µg of trypsin at 37 °C for 18 hours.

#### LC – Tandem Mass Spectrometry Analysis

Tryptic peptides were analyzed by LC-MS/MS on an Ultimate 3000 RSLCnano system (Dionex) coupled to an Orbitrap Elite mass spectrometer (Thermo Fischer Scientific) with a nanospray ion source. Fifteen µL of sample (approximately 5–10 µg of peptides) was loaded onto a C18 trap column for brief desalting and concentration, then separated on a 25 cm C18 analytical column (Picofrit 75 µm ID, New Objective, packed with MagicC18 AQ resin) over a 140 minute, multi-step gradient of increasing organic phase. Each MS/MS experiment consisted of an initial MS1 scan over a mass range of 400–1800 m/z, followed by 10 subsequent data-dependent collision-induced dissociation (CID) fragmentation events of the top 10 most intense +2 or +3 ions from the MS1 spectrum over an acquisition time of 140 minutes.

#### Data Processing and Statistical Analysis

The raw files obtained from LC-MS/MS were converted to mzXML format using MSconvert from the ProteoWizard software^33^. The resulting mzXML files were used to identify proteins by searching against human UniProtKB database on the LabKey server using X!Tandem algorithm^34–36^. Search results from X!Tandem were then analyzed by PeptideProphet and validated using ProteinProphet^37,38^. Protein groups and peptides with a score greater than 0.9 and 0.6, respectively, were retained for protein identification and quantitation. Heavy-to-light (H/L) ratioswere computed for cysteine-containing peptides with acrylamide labels using the Q3 quantitation algorithm^27^. The fragment ion mass accuracy was set to the default ±0.5 Da in X!Tandem. Only peptides with precursor fractional delta mass <20 ppm were used for quantitation. Peptide H/L ratios were averaged for each protein group by each M-LAC fraction resulting in three separate quantitation values for UNB, AAL and PHA. Additionally, global protein levels were calculated as an average of all glycoforms, using all quantitated peptides regardless of which M-LAC fraction the peptide was identified in. Protein groups were subsequently assembled by gene name into normalized gene groups. One gene name was picked to represent each group. H/L ratios of each group were log(2) transformed and median-centered (across all fractions per sample) for normalization. A permutation two-sample t-test (Welch’s unequal variance) was performed on the mean H/L ratios for genes quantified in BPH and PCa groups using perm (R package) to randomly shuffle the data and estimate the distribution of the test statistic^39^. The p-value for the observed test statistic was then determined from the sampling distribution. False discovery rate (FDR) was estimated from the permutation test as the number of false discoveries at or above a given p-value. We used an FDR bound of 1% throughout the manuscript for differential quantification. Permutation tests were only performed on proteins that were quantified in ≥3 samples in both the BPH and PCa groups.

#### Method Reproducibility

The reproducibility of the entire platform described above was accessed using three identical replicates of the reference plasma designed to yield a 1:1 heavy-to-light ratio by combining 1,2,3-^13^C3 heavy-labeled reference plasma with 1,2,3-^12^C3 light-labeled reference plasma. After immunodepletion, reduction, and alkylation/labeling steps, each of the three replicates were separated by M-LAC, RP fractionated, digested, and analyazed by LC-MS/MS as described above. The identical replicates yielded acceptable reproducibility, and the data from these experiments is presented in the supplementary material (see Supplementary Dataset 1 for lists of identified peptides with quantitation, and Supplementary Figures S1 and S2 for spectral count and H/L ratio reproducibility, respectively).

### Data Availability

The datasets generated during and/or analysed during the current study are available from the corresponding author on reasonable request.

## Results

**Figure 1:**
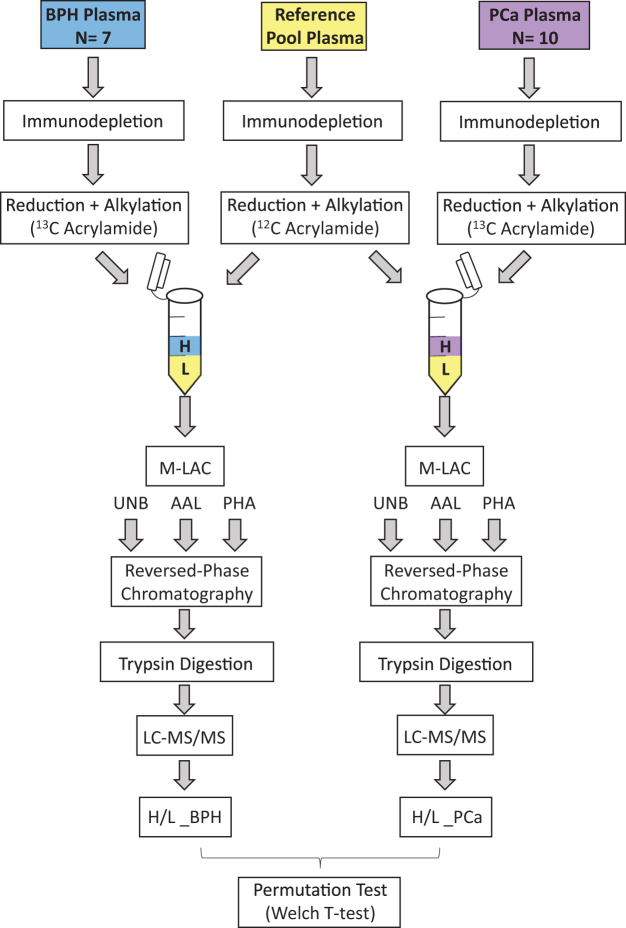
Experimental Design and Analytical Work Flow.

The overall objective of this study was to elucidate differences in the circulating levels of serum protein glycoforms that may have utility in distinguishing between PCa patients from those with BPH. Multi-dimensional chromatographic separation at the intact protein level was used to increase the depth of proteomic analysis given the dynamic range issues associated with blood plasma and serum. Using lectins to fractionate the protein mixture also provides the ability to separate protein glycoforms and to obtain an additional layer of information concerning the types of glycosylation expressed on serum glycoproteins. By using lectins with affinity for particular glycan structures, it is possible to systematically monitor global changes to the levels of specific glycoforms across hundreds of serum proteins from PCa and BPH specimens. Figure 1 shows an overview of the analytical design of these experiments, where all cases (7 BPH samples and 10 PCa samples) were quantitated relative to a pooled reference sample of male plasma that served as an internal control. Each PCa and BPH sample was isotopically labeled with heavy ^13^C-acrylamide, and combined with the reference sample (labeled with a light ^12^C-acrylamide isotope). Each sample was then loaded onto an M-LAC columncontaining AAL and PHA-L/E lectins. AAL preferentially binds fucose linked α1-6 to N-Acetylglucosamine (GlcNAc), and – to a lesser extent – fucose linked α1-3 to N-acetyllactosamine (LacNAc), and was used to enrich for primarily core-fucosylated N-linked glycoforms and to a lesser extent terminal fucosylated N-linked glycoforms^14^. PHA-L has affinity for tri- and tetra-antennary complex-type glycans, while PHA-E recognizes bisected di-, and triantennary complex-type N-linked glycans^14^. These two lectins were used together to enrich for protein glycoforms containing highly-branched, complex-type structures. Three separate fractions were collected from the M-LAC column including the flow-through of unbound proteins (UNB fraction), proteins bound to AAL (AAL fraction), and proteins bound to PHA-L/E (PHA fraction). For each specimen, each of the three M-LAC fractions was then further fractionated by reversed-phase chromatography, trypsinized, and analyzed by LC-MS/MS, as described in detail above. For complete lists of peptide identification and quantitation per fraction, per sample, please see Supplementary Dataset 1. Comparisons were made between PCa and BPH groups among proteins that were quantitated in at least three samples in each group. For a complete list of all protein H/L values in each sample, please refer to Supplementary Dataset 2.

### Depth of Proteomic Analysis

**Figure 2:**
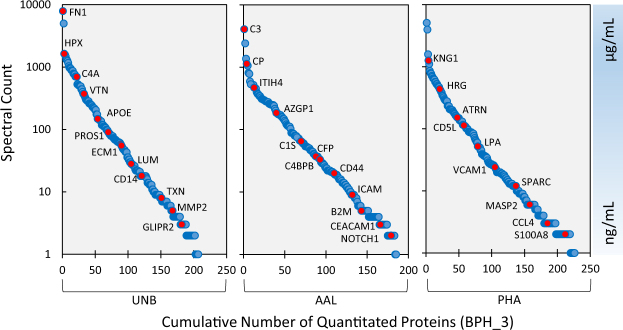
Depth of Proteomic Analysis – the above figure plots the spectral count (per protein) against the cumulative number of quantitated proteins ordered from highest to lowest spectral count (by unique gene name). Examples of the types of proteins that were quantitated are highlighted in red for reference. Literature values of concentrations for these proteins were used to estimate the dynamic range of the protein mixture. This plot was generated from a single, representative sample with an average number of protein identifications. Across all M-LAC fractions, 315 proteins were quantitated in this sample.

Due to the complexity and large dynamic range of protein concentrations inherent to the human serum and plasma proteome, we opted to pre-fractionate the protein mixture to increase lower-abundance protein identifications^10^. The use of high-resolution mass spectrometry also increases the number of identifications and the extent of protein sequence coverage. In this study, samples were extensively fractionated at the intact protein level and separated by glycoform. By LC-MS/MS analysis, on average, 187 proteins (5% FDR, collapsed by gene name, quantified in at least three samples) were quantitated in the UNB fraction, 141 in the AAL fraction, and 159 in the PHA fraction among the PCa specimens. The BPH serum samples yielded 183, 183, and 203 quantitated proteins in the UNB, AAL, and PHA fractions, respectively. These numbers of quantitated proteins results are typical for the chosen methodology, which involves the isotopic labeling of cysteine residues via alkylation with heavy and light isotopes of acrylamide, as described above. A cysteine labeling approach was utilized for efficient labeling at the protein level (versus peptide level) to ensure compatibility with the intact protein fractionation used in our sample preparation. Quantitation is therefore limited to cysteine-containing peptides, which restricts the number of proteins available for quantitation. Furthermore, the reported number of proteins quantitated across samples is a result of restricting proteins to those present in at least three samples in both the BPH and PCa groups to allow for statistical analysis. Figure 2 demonstrates the range of proteins quantitated in a single representative sample (BPH_3, Table 1), plotting spectral counts versus cumulative number of quantitated proteins. Spectral counts and known proteins concentrations in blood were used to estimate the dynamic range of the fractionated protein mixture, which contained proteins with concentrations ranging from micrograms per milliliter (such as C3, VTN, and AZGP1) down to the nanogram per milliliter level (such as MMP2, ICAM1, and CEACAM1)^9,40–42^. A complete list of the number of proteins quantified for each individual sample can be found in Supplementary Dataset 3, and a list of identified peptide sequences can be found in Supplementary Dataset 1.

### Protein Expression Relative to Reference Sample

**Figure 3:**
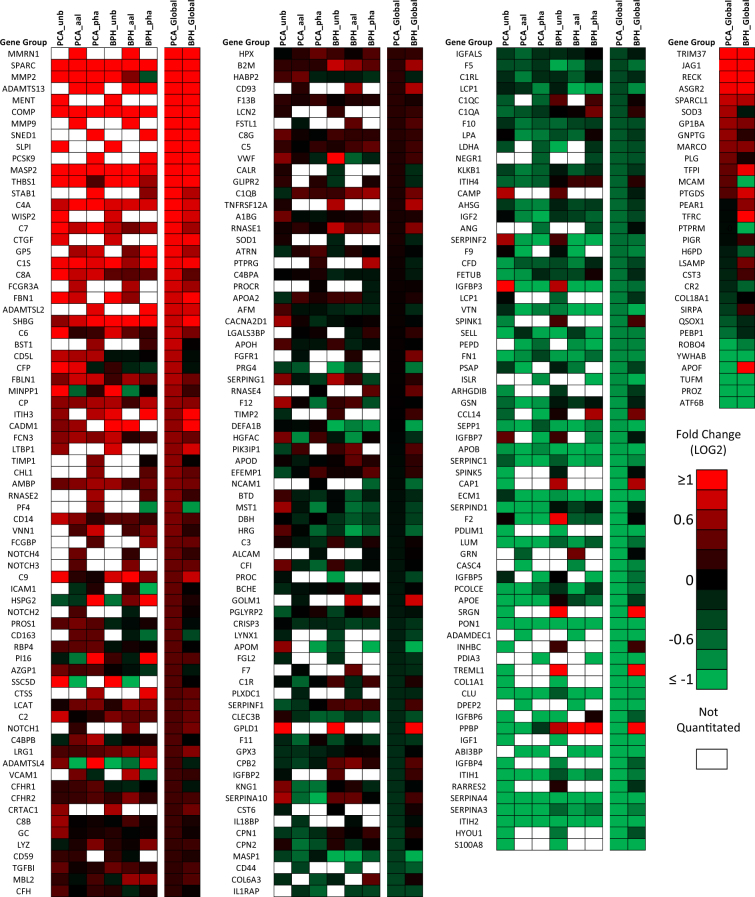
Heat map color-coded by mean fold change (log2H/L) in the PCa and BPH groups with respect to the reference plasma. Each M-LAC fraction is represented in its own column and proteins are ordered from largest fold change in the PCa group at the global protein level. This list of 248 proteins includes those that had an N ≥ 3 for quantitation per M-LAC fraction and/or at the global protein level (far right).

To quantitate alterations in protein abundance, cases (BPH or PCa) were isotopically labeled with heavy 1,2,3 ^13^C-acrylamide. All cases were compared against a reference pool sample isotopically labeled with the light ^12^C-acrylamide isotope. The mass of ^13^C- and ^12^C-acrylamide differs by 3 Daltons, inducing a shift in the mass spectrum of cysteine-containing peptides. The heavy-to-light (H/L) ratio is calculated based on the peak intensity of the heavy and light forms of the peptide integrated across the elution of the peak. An overview of protein quantitation relative to the reference sample is presented in Fig. 3, which features a heat map color-coded by average log2H/L values for each M-LAC fraction in PCa and BPH groups. The proteins are ordered along the horizontal axis from high to low global protein log2H/L values in the PCa group. The set of 248 proteins presented in Fig. 3 includes all quantitated proteins with an n ≥ 3 at the global protein level (i.e. there were at least three samples containing the protein in both groups). Blank (white) spaces in the heat map indicate one of two instances: (1) there were not enough data points (n < 3) in the group to perform statistical analysis, or (2) protein glycoforms were not detected in that fraction in any sample (n = 0). In the former, the H/L data points that were present contributed to the global protein level.

Both PCa and BPH demonstrate deviation from the reference sample, however, the patterns in protein expression were overall quite similar between the BPH and PCa groups. At the global protein level, the most overexpressed proteins (with respect to the reference sample) in the PCa samples were also highly overexpressed in the BPH group, including SPARC, metalloproteinases MMP2 and MMP9, ADAMTS13, MENT, COMP, SNED1, SLPI, and PCSK9. Similarly, proteins with decreased global levels in the PCa group showed comparable decreased levels in the BPH group, including SERPINA4, S100A8, and PON1, among others. The most over-expressed protein in the dataset was MMRN1 (multimerin-1), a glycoprotein with 23 N-linked glycosylation sites that was exclusively detected in the AAL fraction. Global MMRN1 levels were highly elevated in both groups, with a six-fold increase quantitated in the PCA group and a seven-fold increase in the BPH group (PCA log2H/L = 2.59, BPH log2H/L = 2.91, p-value = 0.477). In general, the same trend was found among the protein levels in individual M-LAC fractions. For most proteins, fold-changes were in concordant directions across M-LAC fractions and were similarly expressed with respect to the reference sample in the PCa and BPH groups. Additionally, protein glycoforms were typically identified in the same M-LAC fractions in the PCa and BPH groups. We did not observe any cases in which all of the PCa glycoforms of a given protein were found in one M-LAC fraction and all the BPH glycoforms of that same protein were found in another, which would have clearly indicated a blatant contrast in glycosylation patterns. However, some glycoform-specific alterations were observed that were not immediately apparent at the global protein level. For example, MINPP1 (Fig. 3) was quantitated in all three fractions, but demonstrated elevated levels in the UNB fraction, decreased levels among the AAL glycoforms, and relatively unchanged levels among the PHA glycoforms. The global protein level was slightly overexpressed (less than 1.5-fold), demonstrating which M-LAC fractions were driving the overall trend. This trend, however, was observed in both the PCa and BPH groups, rendering MINPP1 ineffective for distinguishing PCA from BPH. ADAMTSL4, SSC5D, VCAM1, and IGFBP7, to highlight a few, presented similar cases in which different M-LAC fractions showed log2H/L ratios in opposing directions – a trend that would not have been observed without glycosylation-based fractionation – but ultimately resulted in indistinguishable patterns between PCa and BPH groups.

In some cases there were insufficient data points to quantitate glycoforms of individual M-LAC fractions, but had an n ≥ 3 once values from all fractions were combined to determine a global protein level, as is shown in Fig. 3. TRIM37, JAG1, RECK, ASGR2, and SPARCL1 all demonstrated at least 1.5-fold over-expression compared to the reference sample at the global protein level in PCa and BPH groups. Some of the more striking differences were observed in TFRC and APOF, in which the BPH group demonstrated a 2-fold increase, while the PCa group was unchanged (TFRC) or decreased (APOF).

### Differential Expression Between PCa and BPH

**Figure 4:**
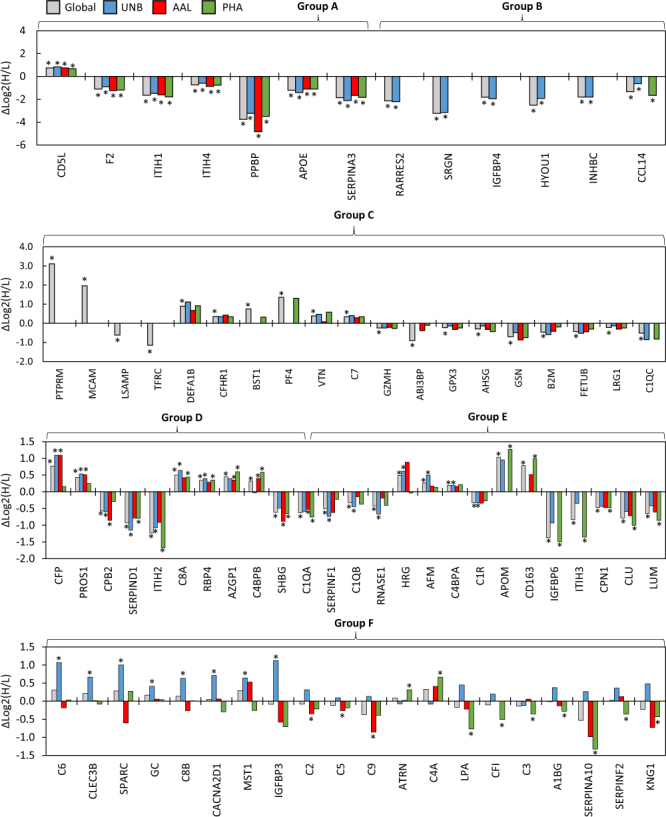
Difference in mean (Δ = log2H/L_PCa − log2H/L_BPH) plotted by M-LAC fraction per protein. Asterisk denotes a statistically significant difference in mean (p ≤ 0.05). Proteins are grouped by expression patterns: (**A**) Quantitated in all fractions and significantly different in all fractions; (**B**) Significantly different in every M-LAC fraction it was quantitated in; (**C**) Only significant at the global protein level; (**D**) Significantly different in multiple M-LAC fractions; (**E**) Significantly different in only one M-LAC fraction and at the global protein level; (**F**) Significantly different in one M-LAC fraction and not at the global protein level.

Among the quantitated proteins shown in Fig. 3, a permutation t-test was performed to determine significant differences between the BPH and PCa log2H/L ratios for those with an n ≥ 3 in each group. A t-test was done at the global protein level, which was calculated as an average of all identified glycoforms, for which 248 comparisons were made. Comparisons were also made between each of the three M-LAC fraction (for proteins with n ≥ 3 within that fraction). Altogether 153 proteins were compared in the AAL fraction, 156 in the PHA fraction, and 168 in the UNB fraction. A complete list of PCa and BPH mean log2H/L values and corresponding p-values can be found in Supplementary Dataset 4. To determine the extent of differential expression in protein levels between PCa and BPH groups, the difference in mean log2H/L was calculated for each protein (Δ = log2H/L_PCa_ − log2H/L_BPH_) and plotted in Fig. 4. This figure illustrates a summary of the direction and magnitude of the difference in log2H/L values in each M-LAC fraction for proteins that had statistically significant differences in one, two, or all three fractions, and/or at the global protein level. Fifty-nine proteins had significantly different levels (p-values ≤ 0.05) at the global protein level. Additionally, 18 were significantly different in the AAL fraction, 30 in the PHA fraction, and 37 in the unbound UNB fraction. As shown in Fig. 4, proteins were organized into groups according to the pattern in which the protein levels were altered. In some instances, proteins yielded increased or decreased levels in the PCa relative to the BPH uniformly across all quantitated glycoforms. Seven proteins were quantitated in all three M-LAC fractions and demonstrated differential expression irrespective of glycosylation, as shown in Group A in Fig. 4. Of these, CD5L was the only protein to have significantly over-expressed protein levels that were higher in the PCa than the BPH in every fraction. Circulating levels of F2, ITIH1, ITIH4, PPBP, APOE, and SERPINA3 were lower in PCa than in the BPH across all glycoforms. Similarly, proteins in Group B (Fig. 4) yielded significantly different protein levels in PCa versus BPH in every fraction that they were quantitated in, but were not detected and/or quantitated in all three fractions. Group C (Fig. 4) contains proteins in which significant differences were only determined at the global protein level, suggesting the trend was only statistically significant when expressed as an average of all detected glycoforms due to the cumulative effect of combining all the data points and increasing the statistical power. Among these, global levels of PTPRM, MCAM, DEFA1B, CFHR1, BST1, PF4, VTN, and C7 were significantly higher in the PCa group than in the BPH group, and LSAMP, TFRC, KLKB1, ABI3BP, GPX3, AHSG, GSN, B2M, FETUB, LRG1, and C1QC were significantly lower in the PCa than in the BPH.

#### Glycoform-specific changes in protein expression

In many cases, glycoform-specific alterations were observed in the patterns of protein expression between the PCa and BPH groups, as is depicted in Groups D, E, and F in Fig. 4. Proteins in Group D were quantitated in all M-LAC fractions and yielded significant differences among multiple glycoforms. Properdin (CFP) showed significantly elevated levels in PCa compared to BPH among core-fucosylated glycoforms bound to AAL and among the other-wise or non-glycosylated forms quantitated in the unbound fraction. Proteins AZGP1, and C4BPB also demonstrated significantly higher levels in glycoforms containing core-fucosylated glycans in the AAL fraction and highly-branched glycans in the PHA fraction, however was not significantly different in the unbound fraction. Notably, the comparison in AZGP1 in the unbound fraction was nearly significant (p = 0.06) and demonstrated a similar trend of increased levels in PCa patients, suggesting that AZGP1 may be over-expressed at the protein level and not necessarily displaying differential glycosylation. However, proteins in Groups E and F were only significantly different in a single M-LAC fraction (proteins in Group E were also significant at the global protein level). For example, proteins such as APOM and CD163 showed significantly increased levels in the PCa group versus the BPH group only in the PHA fraction, however a similar, although insignificant, trend was observed among the glycoforms in the UNB and AAL fractions as well. Interestingly, Group F includes 20 proteins that showed significant differences only in a single M-LAC fraction and were not significant at the global protein level, demonstrating the advantage of fractionating intact proteins by glycoform prior to digestion and MS analysis. Notable in this group is ATRN, which was significantly increased in the PHA fraction exclusively, suggesting that only the highly-branched, complex type glycoforms of this protein were expressed in higher concentrations in PCa patients. Additionally, several members of the complement family yielded lower levels of core-fucosylated glycoforms in PCa patients relative to BPH patients, including C2, C5, and C9. It should be noted that although the difference between the PCa and BPH means were negative in these three proteins (as shown in Fig. 4), all the log2H/L ratios were positive compared to the reference sample, but just to a lesser extent in the PCa group than in the BPH group. This is most notably the case in the AAL fraction of C9, for which the BPH mean was 2-fold higher than the reference sample, and the PCa mean was only 1.11-fold higher than the reference sample (p = 0.024).

In the case of KNG1, the changes in protein level was significantly different in all M-LAC fractions, but not in concordant directions. KNG1 protein levels were lower in the PCa group among glycoforms in the AAL and PHA fractions, but were elevated in the non- or otherwise-glycosylated forms in the UNB fraction. In this case, the opposing trends among the M-LAC fractions resulted in a loss of significance at the global protein level. By fractionating by glycosylation type, we were able to find differences among certain glycoforms, even when the global protein level was unchanged – a trend that would have been missed in proteomic workflows that do not target glycoproteins specifically.

## Discussion

In this study we compare alterations in protein expression in men with BPH relative to normal male plasma to the differential protein expression derived from prostate cancer patients. We hypothesized that differential expression would be observed among protein glycoforms in specific M-LAC fractions and that these differences may show potential for distinguishing BPH from PCa. We observed alterations in circulating levels of proteins both at the global level and among specific glycoforms captured by lectins with affinity for core-fucosylated species (AAL) and highly branched complex-type glycans (PHA-L/E). Quantitation relative to a pooled reference sample revealed substantial similarity in protein and glycoprotein expression among PCa and BPH groups, leaving only a small number of cancer-specific alterations. For this reason, we included proteins with a fold change of ≥1.5 (0.585 on a log2 scale) in our analysis to improve our sensitivity for smaller, more subtle changes in protein level. The extent of overlap in protein expression patterns between these two groups is not entirely unexpected, as it is known that inflammatory and immune response proteins are detectable in the blood of cancer patients in addition to proteins derived from the tumor itself^43^. These results speak to the difficulty in identifying (glyco)proteins that can effectively distinguish PCa from BPH, which is a pitfall of the currently used clinical biomarker for prostate cancer, PSA. Inflammation plays a role in the pathogenesis of both BPH and cancer^43–46^, and indeed a number of inflammation-associated proteins were detected and quantitated within this sample set. Predominating among these were several glycoproteins of the complement system. Notably, complement activators MASP2, or mannan-binding lectin serine protease-2, C1S, and the subsequent pro-inflammatory cleavage product C4A all showed elevated levels in all M-LAC fractions among both PCa and BPH patients^47^. Although considerably increased compared to the reference sample, the abundances of these proteins and their separated glycoforms were similarly altered in both groups and may not serve as ideal cancer-specific markers. Notably, SPARC – a secreted glycoprotein that plays a role in tissue remodeling through interactions with the extracellular matrix – was overexpressed by at least 3-fold with respect to the reference sample in both PCa and BPH across all M-LAC fractions. Although SPARC has been reported to be dysregulated in several different types of cancers^48,49^, has been associated with prostate cancer progression and bone metastasis^50–53^, and has been suggested as a possible target for cancer therapeutics^48,54,55^, the elevated global protein level of SPARC in this study was determined to be nonspecific for prostate cancer. A significant difference between PCa and BPH was observed only in the UNB fraction, in which the levels of non- or otherwise-glycosylated forms of SPARC were significantly higher in the PCa group (PCA log2H/L = 2.58, BPH log2H/L = 1.58, p = 0.0019). Although highly overexpressed in both groups, the levels of SPARC showed trends of being more overexpressed in the PCa group in the PHA fraction and at the global protein level (not significant), and merits further study as a potential biomarker in a larger cohort with greater statistical power.

Despite very similar patterns of protein and glycoprotein expression, a number of notable statistically significant alterations were observed between the two groups that should be highlighted. On the global protein level, there were five proteins that yielded large positive differences between PCa and BPH means (i.e. the mean PCa was greater than that of BPH), were elevated at least 1.5-fold with respect to the reference sample in the PCa group (log2H/L ≥ 0.585), and were significantly different between PCa and BPH (p ≤ 0.05). These five proteins were CFP, BST1, CD5L, C8A, and C7. CD5L levels were increased 1.5-fold in every M-LAC fraction in PCa group and remained relatively unchanged in the BPH group, and yielded one of the most statistically significant differences in the dataset (p = 1.52E-07). Additionally, the increase in global levels of proteins AZGP1, and PROS1 were significantly different between PCa and BPH (p = 0.0003 and p = 2.33E-06, respectively), however it should be noted that AZGP1 and PROS1 were not particularly over-expressed in PCa with respect to the reference sample (both yielded log2H/L values around 0.35, or a 1.25-fold increase), making these proteins less ideal as potential candidates for diagnostic biomarkers.

In the above mentioned proteins, separating by glycoform using lectins did not reveal any differences in glycosylation pattern, although the additional chromatography step may have aided in detecting these moderately-abundant proteins. The real advantage of using an M-LAC approach was made evident when glycoform-specific changes in protein levels were observed between PCa and BPH groups, especially when no changes were evident at the global protein level. These alterations in glycoform expression would be missed without lectin fractionation. The most notable glycoform-specific alterations were observed in the PHA fraction, which contained glycoforms with highly-branched complex type glycans. The degree of branching among N-linked glycan structures has been previously shown to be differentially expressed in cancer, with a reported increase in the expression of complex N-glycans with a β1,6-branched GlcNAc due to the increased activity of acetylglucosaminyltransferase GNT-V^13,24^. Overexpression of tri- and tetra-antennary N-glycan structures has been observed in cell line xenograft mouse models of prostate cancer and has been associated with castration-resistant prostate cancer in human patients^18^. Here, we observe some of the most significant differences in PHA glycoforms in proteins CD163 (p = 0.034), C4A (p = 0.019), and ATRN (p = 0.007), all of which were over-expressed in the PCa group. ATRN (attractin) has been reported as an important predictor of Gleason score and has been proposed as a diagnostic marker in human sera in a study by Cima *et al*. in a genetics-guided proteomic experiment that looked for differential expression of potential glycoprotein biomarkers that were discovered in Pten cKO mice in the sera of men with localized prostate cancer compared to men with BPH^56^. CD163 has also been associated with prostate cancer. Interestingly, CD163 (hemoglobin scavenger receptor M130), is a secreted glycoprotein expressed by tumor-associated type M2 macrophages, and has been reported to be associated with prostatic inflammation and upregulated in prostate cancer with regard to tumor extension, metastasis, and biochemical recurrence^57–60^. In this study, CD163 was only detected and quantitated in the AAL and PHA fractions (no non- or otherwise glycosylated forms), suggesting the presence of core-fucosylation and extensive branching of complex type glycans. Furthermore, CD163 was significantly increased in the PHA fraction of PCa samples compared to BPH, but was only slightly over-expressed compared to the reference sample (PCA log2H/L = 0.373, BPH log2H/L = −0.610, p = 0.034). The CD163 glycoforms in the AAL fraction were also slightly elevated with respect to the reference sample, but were not statistically different from the BPH glycoforms. Given these data, CD163 glycosylation patterns and overexpression merit further investigation in a larger cohort. It should be noted that although AAL and PHA-L/E lectins are widely used, and their specificity is well documented, the exact glycan structure are not elucidated using this method, and additional glycomic or intact glycopeptide experiments would need to be performed to confirm glycan composition^14^. We have previously published a study that profiled intact glycopeptides derived from plasma glycoproteins, and have confirmed the presence of fucosylated glycopeptides were identified for CD163, C4A, and ATRN, and highly branched glycopeptides were identified for C4BPB and AZGP1^61^ in plasma.

Significantly increased expression among core-fucosylated glycoforms was not commonly observed. Most statistically significant difference found in this M-LAC fraction were observed among proteins that were also elevated across all (or most) of the M-LAC fractions, and not to the AAL-bound glycoforms specifically, as shown in the proteins of Group A and D in Fig. 4. Notably, PHA and AAL glycoforms of the protein C4BPB were both elevated relative to the reference sample and were significantly higher in the PCa group than in the BPH group (PHA p = 0.006, AAL p = 0.032), but showed considerable overlap in the log2H/L ratios in the UNB fraction, suggesting increased expression only among these glycoforms. A similar expression pattern was observed in AZGP1.

Although overexpressed proteins arguably make for more definitive measurements in diagnostic biomarkers, this study revealed several proteins that demonstrated decreased expression in the PCa group but striking elevation in the BPH group. Notable among these was the proteoglycan SRGN. SRGN was the most significantly different protein in the UNB fraction, with a three-fold increase in the BPH group relative to the reference sample and a decrease equivalent in magnitude in the PCa group (PCA log2H/L = −1.53, BPH log2H/L = 1.59, p = 0.003). SRGN, or serglycin, has eight o-linked sites carrying glycosaminoglycans and no N-linked glycosylation sites (SRGN was identified exclusively in the UNB fraction). Serglycin has been described as being at the crossroads of inflammation and malignancy, playing a vital role in immune response through interactions with chemokines, cytokines and growth factors^62^. Interestingly, SRGN was only overexpressed in the BPH group in this study, and markedly decreased in the PCa group, suggesting it may be useful in positively identifying cases of inflammation associated with BPH.

In summary, we successfully quantitated hundreds of glycoproteins from PCa and BPH patients to interrogate any differential expression between these two groups. Our approach combined traditional bottom-up proteomics with a series of chromatographic separations to improve the depth of analysis and to systematically screen a complex mixture of proteins for global changes in specific types of glycosylation. Using a multi-lectin approach, our method proved to be useful in separating and quantitating specific protein glycoforms, and yielded an information-rich dataset. By isotopically labeling cysteine-containing proteins, we were able to determine differences in protein expression between the PCa and BPH groups relative to a normal reference sample. Global patterns of differential expression of core-fucosylated or highly-branched glycoforms across the quantitated fraction of the serum proteome were not consistently observed as they have been previously described in glycomic studies that remove the glycan from the protein. In keeping the glycan conjugated to the protein and thus retaining protein-specific information, the breadth of the analysis is subject to the dynamic range and sampling issues associated with mass spectrometry-based proteomics. Upon removing glycans, the differential expression of specific glycan types are cumulative and more readily observed. In this study, we identified alterations among the AAL and PHA glycoforms that were localized to specific, individual proteins rather than observing any larger systemic difference in glycosylation. The expression of core-fucosylation and highly-branched glycoforms was increased in some proteins and decreased in others, with no discernable trend to differentiate the PCa and BPH groups. Among considerably similar proteomic and glycoproteomic signatures, a number of proteins showed significant differences irrespective of glycosylation, including CD5L, CFP, C8A, C7, and BST1. Additionally, statistically significant glycoform-specific alterations were observed among M-LAC fractions, including SPARC in the UNB fraction, CD163, C4A and ATRN in the PHA fraction, and C4BPB and AZGP1 in both the AAL and PHA fractions. These alterations in protein expression and differential glycosylation patterns may warrant further study in a larger cohort to investigate the reproducibility of our findings, however, a clinically useful biomarker would ideally yield a more dramatic fold-change between PCa and BPH than was observed in any proteins in this study. A strategy for future validation of any glycoproteins of interest may include the use of antibody-lectin assays built to target specific protein glycoforms, such as those use by Sinha, J. *et al*.^63^. Additionally, further experiments for analysis of intact glycopeptides may also provide more detailed annotation of protein glycosylation status and reveal more specific alterations in glycan structure between PCa and BPH, as lectins, although useful tools for separation, provide limited information regarding glycan structure.

## Electronic supplementary material

Table of Contents and Figures

Dataset 1A

Dataset 1B

Dataset 1C

Dataset 1D

Dataset 1E

Dataset 1F

Dataset 1G

Dataset 1H

Dataset 1I

Dataset 1J

Dataset 1K

Dataset 1L

Dataset 1M

Dataset 1N

Dataset 1O

Dataset 1P

Dataset 1Q

Dataset 1R

Dataset 1S

Dataset 1T

Dataset 2

Dataset 3

Dataset 4
