# Supplementary material for: Multi-lectin Affinity Chromatography and Quantitative Proteomic Analysis Reveal Differential Glycoform Levels between Prostate Cancer and Benign Prostatic Hyperplasia Sera
Source: Sci Rep. 2018 Apr 25;8:6509. doi: 10.1038/s41598-018-24270-w (PMC5916935; doi:10.1038/s41598-018-24270-w)

SUPPLEMENTARY INFORMATION FOR

Multi-lectin Affinity Chromatography and Quantitative Proteomic Analysis Reveal Differential Glycoform Levels between Prostate Cancer and Benign Prostatic Hyperplasia Sera

Sarah M. Totten[1], Ravali Adusumilli[1], Majlinda Kullolli[1], Cheylene Tanimoto[1], James D. Brooks[2], Parag Mallick[1], Sharon J. Pitteri[1*]

(1) Canary Center at Stanford for Cancer Early Detection, Department of Radiology,

Stanford University School of Medicine, Palo Alto, CA, 94304

(2) Department of Urology, Stanford University School of Medicine, Stanford, CA 94305

*Corresponding Author:

Sharon J. Pitteri

Canary Center at Stanford for Cancer Early Detection, Department of Radiology, Stanford University

School of Medicine, 3155 Porter Drive MC5483, Palo Alto, CA 94304

spitteri@stanford.edu

Phone: 650-723-6076

SUPPLEMENTARY INFORMATION

TABLE OF CONTENTS

Supplementary Dataset 1

A-C: Peptide Identifications in Replicates 1-3, respectively

D-J: Peptide Identifications in Samples BPH_1-7, respectively

K-T: Peptide Identifications in Samples PCa_1-10, respectively

Supplementary Dataset 2

Complete list of protein quantitation per M-LAC fraction, per Sample

Supplementary Dataset 3

Number of Quantitated Proteins per sample

Supplementary Dataset 4

A: Summary Statistics of Significant Proteins, with p-values

B: Summary Statistics of Non-significant Proteins, with p-values

Supplementary Figure S1

Histogram of protein Log2(H/L) values across all fractions (A), and in the UNB (B), AAL (C), and PHA (D) fractions in identical reference/reference replicates. H/L ratios were Log2 transformed and median-centered for normalization, as described in the methods section of the main text. As expected, the distribution of values form a narrow Gaussian curve centered around Log2(H/L)=0 (1:1 ratio).

Supplementary Figure S2

Protein spectral count agreement between identical reference/reference replicates, by M-LAC fraction. Inset is the linear fit equation and R2 value. All spectra (quantitated and not-quantitated) with PeptideProphet scores ≥0.6 were counted.

Supplementary Figure S1. Histogram of protein Log2(H/L) values across all fractions (A), and in the UNB (B), AAL (C), and PHA (D) fractions in identical reference/reference replicates. H/L ratios were Log2 transformed and median-centered for normalization, as described in the methods section of the main text. As expected, the distribution of values form a narrow Gaussian curve centered around Log2(H/L)=0 (1:1 ratio).

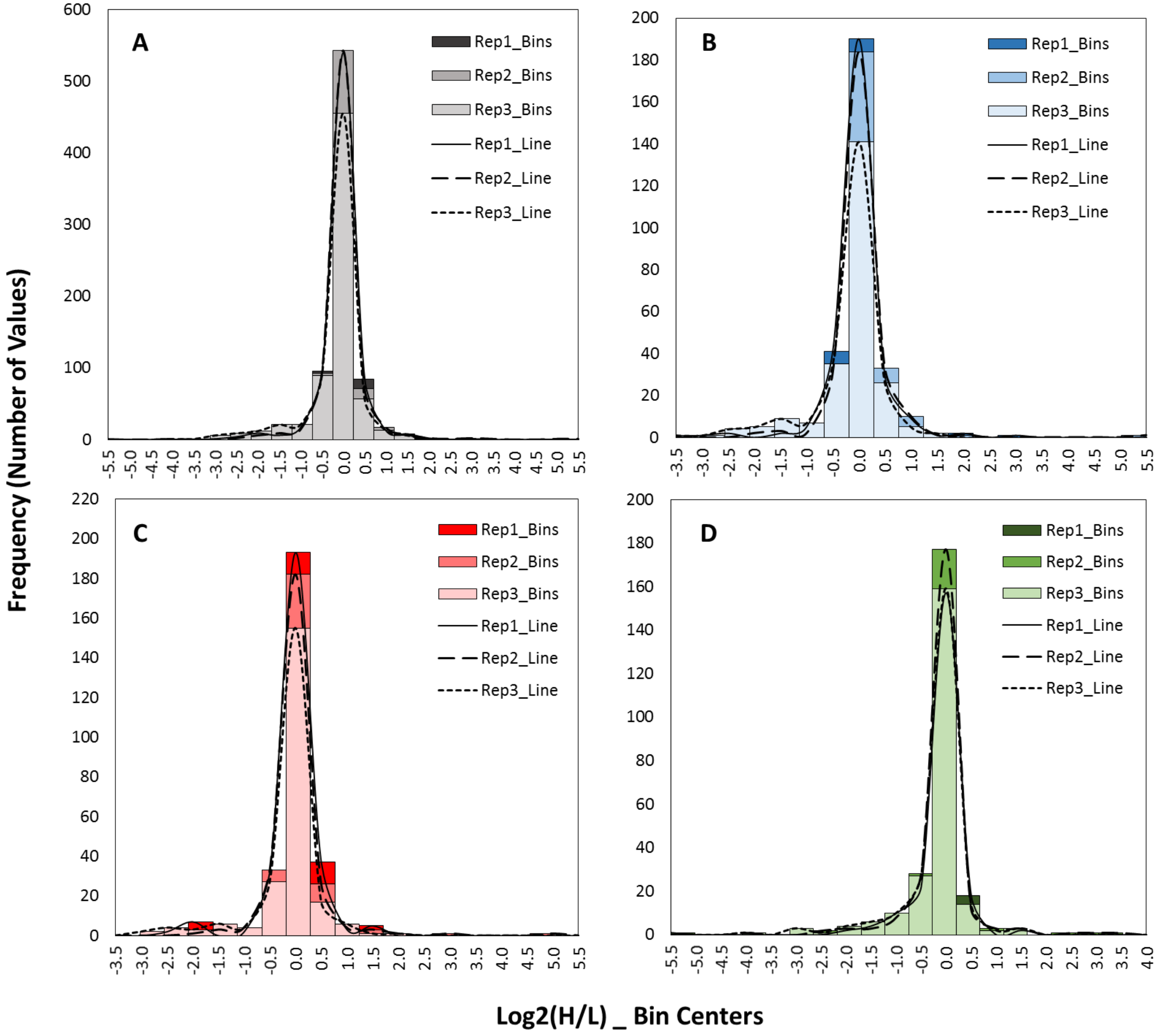

Supplementary Figure S2. Protein spectral count agreement between identical reference/reference replicates, by M-LAC fraction. Inset are the linear fit equation and R^2 value. All spectra (quantitated and not-quantitated) with PeptideProphet scores ≥0.6 were counted.

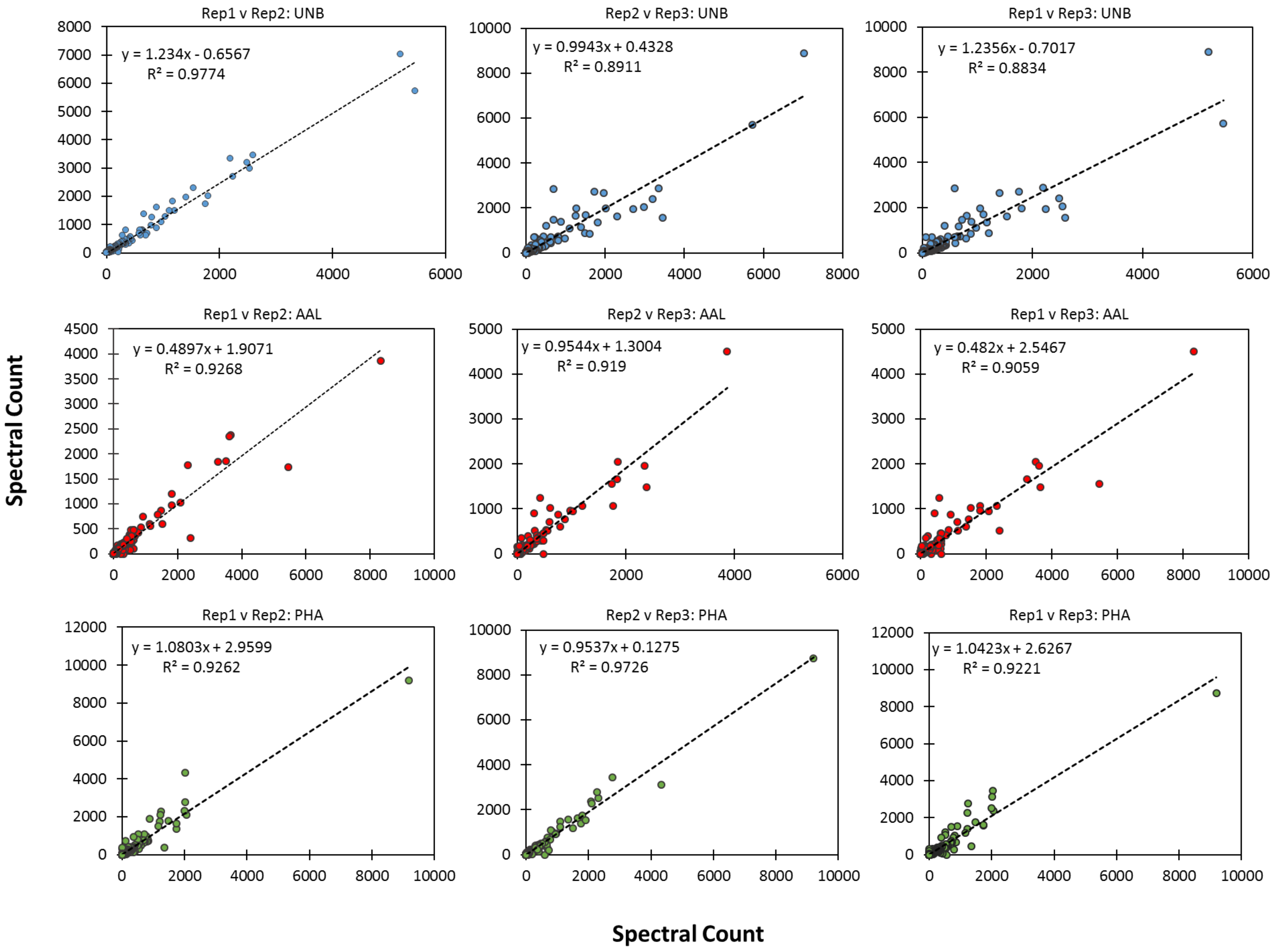

Supplement: Supplementary file 1 — Table of Contents and Figures [file 41598_2018_24270_MOESM1_ESM.pdf]
